# The Gratwick Research Laboratory

**Published:** 1906-09

**Authors:** 


### The Gratwick Research Laboratory.

SOME years ago, through the liberality of Mrs. W. H. Gratwick, of this city, a fine building was constructed on High street opposite Buffalo General Hospital, for the purpose of housing the cancer laboratory which has been conducted the past few years by Dr. Roswell Park as a part of the medical department of the University of Buffalo, and lately has been placed under the supervision of the state department of health. Dr. Gaylord and Dr. Clowes have conducted experiments in this laboratory relating to the cause of cancer that have attracted attention all over the world.

Before the adjournment of the last legislature, at the instance of Senator Henry W. Hill, an appropriation of $18,000 was made by the state for the purpose of continuing the work during the current fiscal year. On June 2, 1906, Mr. Charles A. Ballance, of London, spent the day in Buffalo and visited the Gratwick laboratory in company with Dr. A. Vander Veer, of Albany. Mr. Ballance and his brother, Mr. H. A. Ballance of Norwich, Eng., were Dr. Vander Veer's guests during the meeting of the American Surgical Association at Cleveland and spent a day in Buffalo en route, the Messrs. Ballance being prominent surgeons in their respective homes.

Soon after his return to England, Mr. Ballance, senior, who has had a large experience in cancer research, hence is competent to judge of the value of the work being conducted in Buffalo, addressed a letter to Senator Hill relating thereto, which we deem of sufficient interest and importance to publish. The letter is as follows:

July 11, 1906.
106 Harley St. Cavendish Square, W. London.

"*My Dear Sir*—I have recently had the privilege of visiting the cancer laboratory of the University of Buffalo and I should like to tell you what I think of the work going on there. In no part of the world is better work being done to unfold the intricate problems surrounding the intimate nature of cancer. I was myself engaged for many years in cancer research and so feel competent to form a judgment on the work being carried out at Buffalo. In a disease like diphtheria, which kills quickly, say in three or four days, it was possible, with the advance of bacteriology and chemical pathology, soon to discover its real cause and devise a serum remedy for its treatment. In cancer we have a disease which runs a much longer course—one, two or three years—in the human body and, therefore, we cannot expect either to find the real cause or happen upon its successful treatment without prolonged research, a research which may result in say 25 years in great triumph over human misery and pain. It is to Buffalo that I shall look, year after year, for those discoveries, which will relieve mankind from the dread spectre of this terrible disease. There is no work in all the world more deserving of ample support than the cancer research laboratory.

"I remain, my dear sir,
Yours very truly,
CHARLES A. BALLANCE."

If there were a doubt in the minds of any persons as to the propriety of annual appropriations by the state for scientific medical work on the lines conducted in the Gratwick laboratory, this letter of Mr. Ballance should be sufficient answer to such objections. No country in the civilised world spends as little money from the public treasury for scientific medical investigation as the United States. Mr. Ballance has rendered great service in placing his approval upon the investigations going on in Buffalo relating to the cause of cancer.

---

ATTENTION is invited to advertising page x, whereon the books, instruments and appliances, of the late Dr. Showerman, Batavia, are offered for sale. Any physician in need of such articles will find it advantageous to consult Dr. Harry R. Trick, 1195 Main Street, Buffalo, of whom all information may be obtained.

---

---

DR. SAINT CLAIR MCKELWAY, editor of the Brooklyn Eagle, is among the Americans of distinction now in England. Dr. McKelway is vice-chancellor of the University of the State of New York, and will represent that institution at the 400th Anniversary of Aberdeen University to be held about the middle of September.

